# The Regulatory T Cell in Active Systemic Lupus Erythematosus Patients: A Systemic Review and Meta-Analysis

**DOI:** 10.3389/fimmu.2019.00159

**Published:** 2019-02-18

**Authors:** Wenli Li, Chuiwen Deng, Hanbo Yang, Guochun Wang

**Affiliations:** ^1^Department of Rheumatology, China-Japan Friendship Hospital, Beijing, China; ^2^Department of Rheumatology and Clinical Immunology, Peking Union Medical College Hospital, Chinese Academy of Medical Sciences and Peking Union Medical College, Beijing, China

**Keywords:** regulatory T cells, Foxp3, systemic lupus erythematosus, autoimmunity, meta-analysis

## Abstract

**Background:** Regulatory T cells (Tregs) researches in systemic lupus erythematosus (SLE) have floundered over the years, reports on the numbers and function of Tregs in SLE present quite contradictory results. We therefore conducted a meta-analysis to verify the changes of Tregs in active SLE.

**Methods:** We systematically searched PubMed, Embase, and ISI web of knowledge databases for eligible articles. In total, 628 active SLE patients and 601 controls from 18 studies were included. Due to a high degree of heterogeneity, a random effects model was used to assess the mean differences in Treg percentages, absolute numbers, and suppression capacities of Tregs between active SLE and controls. Further, subgroup analysis was performed to identify potential sources of heterogeneity.

**Results:** The pooled percentages of Tregs in active SLE patients were found to be lower than those in controls (−0.864 ± 0.308, *p* = 0.005), with great heterogeneity (*I*^2^ = 95.01). The discrepancy of published results might result from the following differences among studies: gating strategies for Tregs, diagnostic criteria for SLE, and thresholds of SLEDAI chosen to differentiate between active and inactive SLE. In active SLE, Tregs gated based on CD25 alone showed lower pooled frequency than those gated by Foxp3^+^ or CD127^low/∅^. The percentages of Tregs in active SLE was significantly lower than that in controls when the enrolled SLE patients were diagnosed according to the 1997 modified criteria, whereas they were comparable to controls when diagnosed by the 1982 criteria; the higher threshold of SLEDAI score used to define active SLE tended to achieve a lower percentage of Tregs. The pooled absolute numbers of Tregs in active SLE were significantly decreased compared to those in controls (−1.328 ± 0.374, *p* < 0.001), but seemed to be unaffected by gating strategies. Suppression capacities of Tregs from active SLE patients showed no abnormalities based on the limited pooled data. Longitudinal monitoring of active SLE showed a significant decrease in Treg percentage at remission.

**Conclusions:** This study implies that loss of Tregs may play a role in the pathogenesis of active SLE and help clarify contradictory Treg results in SLE.

## Introduction

T regulatory cells (Tregs), a subset of T cells expressing the cytokine IL-2 receptor α-chain (CD25), were first identified by their ability to prevent the occurrence of systemic autoimmune diseases in thymectomized mice in the mid-1990s. Accumulating data in recent years indicate that these cells are pivotal for maintaining self-tolerance by suppressing the activation and expansion of auto-reactive lymphocytes through cell-cell interactions or by secreting anti-inflammatory cytokines such as IL-10 and TGF-β (1, 2).

According to the developmental origin, Tregs are broadly classified as tTreg cells (tTregs) derived from the thymus and iTreg cells (iTregs) induced in peripheral tissues. tTregs develop from CD4^+^CD8^+^ thymocytes in the thymus, and the process is guided by T cell receptors (TCRs) that recognize self-peptide-major histocompatibility complex (MHC) complexes. tTregs were proposed to control immune homeostasis and autoimmune responses by controlling the tolerance to self-antigens (3, 4). iTregs, on the contrary, are developed from CD4^+^Foxp3^−^ T cells in the periphery and present clones with TCRs specific for non-self antigens derived from food, bacteria, and other pathogens (5, 6). However, increasing studies suggest that tTregs and iTregs could induce peripheral tolerance to both self and foreign antigens (7, 8). However, it is still not completely known whether there are reliable markers to distinguish these two Treg subsets and changes in the frequencies of tTregs and iTregs. tTregs, but not iTregs, were reported to highly express the transcription factors Helios and Neuropilin-1 (Nrp-1), which exerted positive control of Treg suppressive function and lineage stability (9, 10). Subsequent research also showed high expression of Helios and Nrp-1 in iTregs (11). Thus, so far, no definite phenotypic markers have been identified to distinguish between these two Treg populations (12). An effective method to confirm the origin of Tregs is to analyze their TCR repertoire by deep sequencing (8, 13, 14).

The number and function of Tregs can be regulated by related signaling pathways; for example, it has been verified that the IL-21-driven mechanistic target of rapamycin (mTOR) activation blocks the development of Tregs and underlies the dysfunction of Tregs in SLE (15); In addition, type 1 sphingosine-1-phosphate receptor (S1P1) signaling negatively controls the thymic generation and suppressive function of tTregs, depending on the Akt–mTOR axis (16). The S1P1-mTOR axis also inhibits iTreg generation and maintenance (17).

Currently peripheral Tregs in humans are usually identified by their high expression of membrane CD25 and intracellular forkhead box P3 (Foxp3). As a transcription factor, Foxp3 is essential for the development, stability, and function of Tregs (18). The absence of membrane CD127 is also used as an alternative to Foxp3 to isolate live cells for functional tests (19). However, these markers are also found in some activated T effector cells. Thus, it is challenging to find a unique phenotypic marker for Tregs in humans.

Systemic lupus erythematosus (SLE) is an inflammatory, multisystem, heterogeneous autoimmune disorder characterized by a multitude of autoantibody production and immune complex deposition, causing damage to multiple organs. Dysfunction of T and B cells are believed to be critical factors involved in the pathogenesis of disease (20, 21). As a classical prototype of systemic autoimmune disease, a lack of Tregs or defect in Treg function is generally considered to favor SLE pathology. Thus, correction of defects in either the number or function of Tregs may have great therapeutic effects. Indeed, Treg-based immunotherapies have promising applications for SLE. For instance, adoptive transfer of exogenously expanded Tregs delays disease progression and reduces mortality in murine lupus models (22); some studies have reported that therapies targeting Tregs are of great importance in SLE. For example, Lai et al. confirmed that N-acetylcysteine could improve the disease activity of SLE by blocking mTOR in Tregs (23). Moreover, CD4^+^CD25^+^FoxP3^+^ Tregs were expanded when treated for 12 months with sirolimus, which is associated with a progressive improvement in the disease of active SLE patients (24).

Despite these evidences, we have less confidence in the possible beneficial effects of therapeutic Tregs in SLE patients. Application of Treg-based therapeutic approaches in SLE should be based on the premise that a reduced amount and/or impaired suppressive function of Tregs is implicated in SLE pathogenesis. Nevertheless, studies on the numbers of Tregs in active SLE vs. normal patients present rather contradictory results; reduced (25–34), unchanged (35), or even increased (36–39) frequencies of Tregs have been reported in SLE patients. Importantly, the function of Tregs in SLE also remains controversial (29, 36, 37).

It is conceivable that strategies for quantifying Tregs seem crucial for drawing conclusions about this T cell subpopulation. In addition, differences in patient recruitment (region, diagnostic criteria, treatment status, disease activity, organ involvement) may also account for the apparent discrepancies found in literature. However, to our knowledge, there has been no research to establish the source of these inconsistent results.

Given the fact that the quantitative and qualitative changes of Tregs in SLE are still unclear and that Treg-based immunotherapies show promising therapeutic potency, we performed this meta-analysis to obtain pooled quantitative and qualitative changes of Tregs in active SLE, to establish the source of inconsistent results, and thus gain a more detailed understanding of the role of Tregs in SLE pathogenesis.

## Methods

### Search Strategy

A literature search was conducted in Pubmed, Embase, and ISI web of knowledge databases using the following terms: “regulatory T cell,” “Treg,” “CD4^+^CD25^+^ T cell,” combined with “systemic lupus erythematosus.” Reviews were excluded and only articles written in English were accepted. There were no limits on ethnicity and geographical location. Related references cited in eligible articles were also included, and all documents were updated to August 2018.

### Eligibility Criteria

Studies that fulfilled the following criteria were included: (1) case-control study; (2) evaluating the levels of Tregs in SLE patients; (3) levels of Tregs were presented as ratio of Tregs to CD4^+^ T cells (%), or the absolute number of Tregs (cells/mm^3^); (4) mean (standard deviation /standard error) or median (range/interquartile range) were provided. Conference abstracts that were not published as full-length articles were not included.

### Data Extraction

Data were recorded from eligible articles by two independent researchers. Disagreements were resolved by discussion. The related information included the country where the authors performed the studies, diagnostic criteria, definition of Tregs, treatment status of the recruited participants, threshold of SLEDAI chosen to define active SLE, the number of patients, the frequency of Tregs (%), the absolute number of Tregs (cells/mm^3^), and the suppression percentage of Tregs (%) *in vitro*. When the studies reported standard errors instead of standard deviations, the standard deviation was calculated by multiplying the standard error with the square root of the sample size. When the studies provided medians and ranges (or interquartile ranges) instead of means and standard deviations, we calculated the means and standard deviations by estimation methods (40, 41). The NEWCASTLE-OTTAWA SCALE (NOS) was used to evaluate the quality of included studies.

### Statistical Analysis

Heterogeneity was assessed using the *I*^2^-statistic. *I*^2^ values of 25, 50, and 75% were used as evidence of low, moderate, and high heterogeneity, respectively. The pooled results were obtained using a random effects model when the heterogeneity was high, and a fixed-effects model should be used when the heterogeneity is low or absent. Additional analyses including subgroup analyses and publication bias were also performed to explore the heterogeneity. Sensitivity analyses were conducted to test the robustness of the original results. Meta-analysis was performed using Comprehensive Meta Analysis Version 2.0 software (Englewood, USA). This meta-analysis was performed according to the PRISMA guidelines.

## Results

### Literature Search

**Figure 1:**
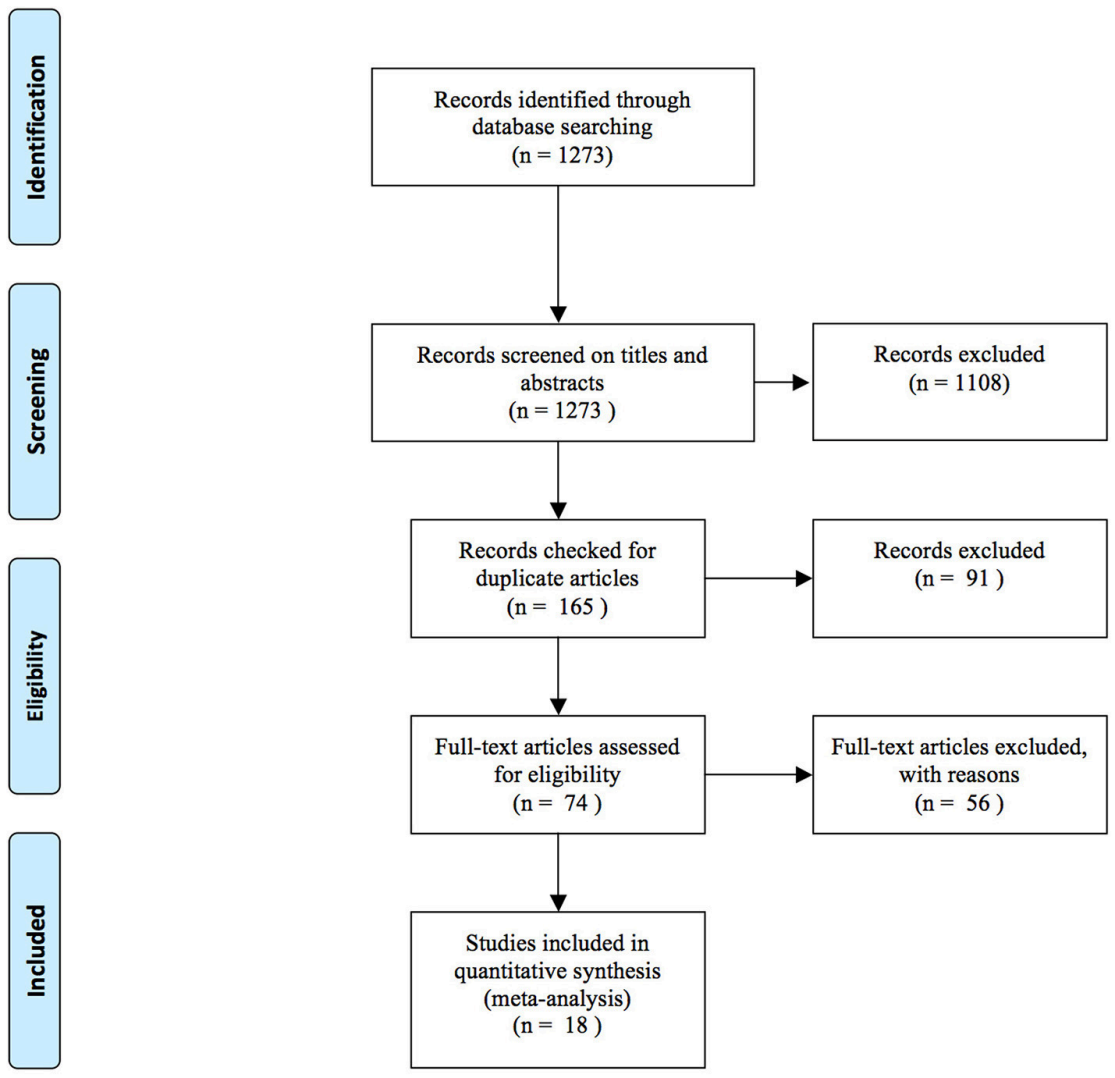
Flow chart of studies included in the meta-analysis.

There were 1,273 potentially eligible articles searched from the databases. A flow chart of the screening process for the articles is shown in Figure 1. A total of 1,108 articles were excluded by screening the titles and abstracts. Then, 91 duplicate articles were excluded: 2 articles were not designed to detect the changes of Tregs in controls, 14 articles did not provide data, and 40 articles were not related to our objective. In total, 18 studies were included in this meta-analysis (25–39, 42–44) (Figure 1).

### Study Characteristics

All characteristics of the included studies are listed in Table 1. This analysis included 628 active SLE patients and 601 controls pooled from 18 eligible studies. Among the studies, five were carried out in China (27, 28, 30, 32, 37), two in Austria (29, 42), three in Brazil (35, 39, 44), one in France (25), one in Hungary (26), one in Germany (36), one in Egypt (31), one in Iran (43), one in Greece (33), one in Poland (34) and one in Indonesia (38). NOS assessment indicated that the eligible studies were of moderate quality.

**Table 1:** Characteristics of studies included in the meta-analysis.

**References**	**Region**	**Diagnosis criteria**	**Treatment status**	**Threshold of SLEDAI for active SLE**	**Treg definition**	**Case**	**Control**	**Tregs in case**	**Tregs in control**	**NOS score**
						**(*n*)**	**(*n*)**	**(mean ± SD,%)**	**(mean ± SD,%)**	
Miyara et al. (25)	France	1982&1997	Treated	>3	CD4^+^CD25^high^	45	82	0.570 ± 0.24	1.290 ± 0.380	5
Barath et al. (26)	Hungary	1997	Treated	≥5	CD4^+^CD25^high^	19	41	3.270 ± 1.880	4.260 ± 1.010	3
Hu et al. (27)	China	1997	Not report	Not report	CD4^+^CD25^+^	20	16	12.920 ± 7.090	53.900 ± 4.700	3
Venigalla et al. (36)	Germany	1982	Treated	>3	CD4^+^CD25^high^FoxP3^+^	14	19	2.650 ± 1.500	1.750 ± 0.440	3
Lu et al. (28)	Taiwan	1997	Untreated	>3	CD4^+^CD25^+^	12	20	3.680 ± 1.890	10.220 ± 7.420	4
Bonelli et al. (29)	Austria	1982	Treated	≥6	CD4^+^CD25^high^	5	24	0.960 ± 0.180	2.000 ± 0.490	4
Yan et al. (37)	China	1997	Untreated	>3	CD4^+^CD25^+^FoxP3^+^	15	15	9.110 ± 2.830	4.780 ± 1.670	3
Bonelli et al. (42)	Austria	1982	Treated	≥6	CD4^+^FoxP3^+^	6	7	16.350 ± 3.800	6.500 ± 1.300	4
					CD4^+^CD25^high^	6	6	0.630 ± 0.080	1.800 ± 0.160	
Yang et al. (30)	China	1982&1997	Treated	≥6	CD4^+^CD25^+^CD127^−^	25	15	4.490 ± 1.430	9.440 ± 2.620	4
Atfy et al. (31)	Egypt	1988	Not report	Not report	CD4^+^CD25^+^	12	10	14.970 ± 6.600	21.300 ± 5.000	4
					CD4^+^CD25^high^			5.900 ± 1.900	8.070 ± 2.040	
					CD4^+^CD25^high^FoxP3^+^			2.900 ± 1.050	4.700 ± 1.200	
Suen et al. (32)	China	1997	Treated	>3	CD4^+^CD25^high^FoxP3^+^	58	36	0.610 ± 0.410	0.860 ± 0.390	4
Henriques et al. (35)	Brazil	1997	Treated	≥5	CD25^high^CD127^low/∅^FoxP3^+^	15	15	8.100 ± 3.700	7.100 ± 2.700	3
Habibagahi et al. (43)	Iran	1997	Treated	≥6	CD4^+^CD25^high^	34	30	1.780 ± 1.120	3.690 ± 1.170	5
					CD4^+^FoxP3^+^			2.242 ± 1.489	3.887 ± 1.061	
Mesquita et al. (44)	Brazil	1982	Treated	Not report	CD25^high^CD127^low/∅^FoxP3^+^	26	26	0.940 ± 0.380	0.660 ± 0.500	4
					CD25^+^CD127^low/∅^FoxP3^+^			1.400 ± 0.800	1.130 ± 0.590	
					CD4^+^CD25^high^			5.200 ± 5.700	1.730 ± 0.800	
					CD4^+^CD25^+^			13.600 ± 9.200	8.000 ± 2.100	
Tselios et al. (33)	Greece	1982&1997	Treated	≥6	CD4^+^CD25^high^FoxP3^+^	61	20	0.854 ± 0.293	1.490 ± 0.190	
Zabinska et al. (34)	Poland	Not report	Treated	≥6	CD4^+^CD25^+^FoxP3^+^	40	19	1.073 ± 0.593	3.327 ± 0.519	
Handono et al. (38)	Indonesia	ACR^*^	Not report	>3	CD4^+^CD25^+^FoxP3^+^	62	62	2.300 ± 2.100	0.900 ± 0.800	4
Mesquita et al. (39)	Brazil	1997	Not report	Not report	CD4^+^CD25^+^CD127^low^	17	10	4.548 ± 2.503	3.008 ± 1.511	3

* *ACR without detailed description*.

### Meta-Analysis of the Treg Percentages in Active SLE Patients

**Figure 2:**
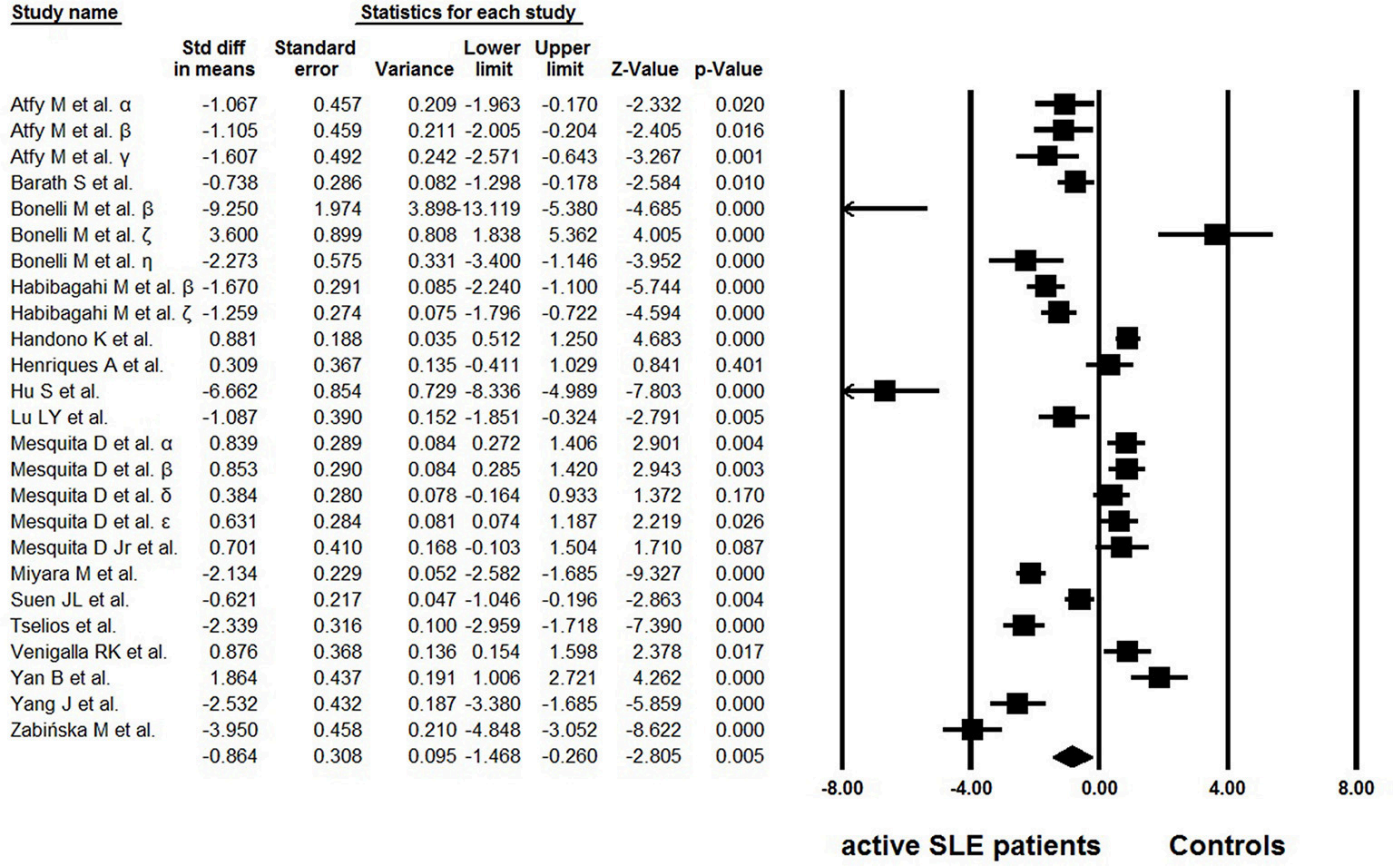
Forest plot of the percentage changes of Tregs in active SLE patients compared with the controls. α: Tregs were gated by CD4^+^CD25^+^; β: Tregs were gated by CD4^+^CD25^high^; γ: Tregs were gated by CD4^+^CD25^high^FoxP3^+^; δ: Tregs were gated by CD25^+^CD127^low/∅^FoxP3^+^; ε: Tregs were gated by CD25^high^CD127^low/∅^FoxP3^+^; ζ: Tregs were gated by CD4^+^FoxP3^+^; η: data from Bonelli et al. (29).

Of the 18 eligible studies, 10 reported lower percentages of Tregs in active SLE than those in the controls (25–34), four articles reported increased percentages (36–39), and no statistically significant difference was found between the groups in one study (35). In addition, three studies analyzed different Treg phenotypes simultaneously, and yielded conflicting results (42–44). High heterogeneity (*I*^2^ = 95.01) was observed between the studies and a random effects model was used in the meta-analysis. In the overall analysis, the percentages of Tregs in active SLE were significantly lower than those in controls (−0.864 ± 0.308, *p* = 0.005, Figure 2).

### Subgroup Analysis and Publication Bias

Considering that different gating strategies of Tregs, enrolled regions, diagnostic criteria, treatment status, threshold of SLEDAI chosen for active SLE definition, and organ involvement are potential elements that might induce bias in the results, subgroup analysis was performed based on these factors.

There were several phenotypes in the recruited articles, and patients could be divided into two groups: earlier sorting strategy only based on CD25 (CD4^+^CD25^+/high^) (25–29, 31, 42–44) and a new strategy with Foxp3 or CD127 staining (FoxP3^+^ or CD127^low/Ø^) (30–39, 42–44). As expected, a significant difference was found between the two groups (*p* = 0.024). The percentages of Tregs were significantly lower in active SLE than those in controls when only based on CD25 (−1.672 ± 0.472, *p* < 0.001). However, the percentages of Tregs in active SLE were comparable to those in the controls when they were gated based on FoxP3 or CD127 (−0.269 ± 0.404, *p* = 0.505).

For subgroup analysis of the diagnostic criteria, three articles were excluded, as two articles did not report the criteria clearly (34, 38), and another paper was the only study that used the 1998 ACR criteria (31). Therefore, Treg frequencies were only compared between researches that applied the 1982 ACR criteria (29, 36, 42, 44) and those that applied the 1997 ACR criteria (26–28, 32, 35, 37, 39, 43). The results showed that the studies recruiting active SLE patients using ACR 1997 were prone to detect lower Treg percentages in active SLE than those in controls (−0.835 ± 0.384, *p* = 0.030), whereas the levels of Tregs in active SLE were comparable to those in controls using the 1982 revised criteria (0.247 ± 0.432, *p* = 0.568); so, these significantly different diagnostic criteria may introduce heterogeneity.

In the recruited studies, only 14 studies provided the treatment information clearly: the enrolled patients were untreated in two researches (28, 37), and treated in other studies (25, 26, 29, 30, 32–36, 42–44). The difference was not statistically significant between the two groups (0.375 ± 1.056 vs. −0.865 ± 0.367, *p* = 0.267).

We next questioned whether the threshold of SLEDAI score chosen to define active SLE could also be a source of discrepancy. Six studies defined active SLE with SLEDAI ≥ 6 (29, 30, 33, 34, 42, 43), 2 studies defined active SLE with SLEDAI ≥ 5 (26, 35) whereas 6 studies defined active SLE based on SLEDAI ≥ 3 (25, 28, 32, 36–38). Interestingly, a significant difference in Treg frequency was found between these groups, and it seems that the higher threshold of SLEDAI score applied tended to obtain a lower percentage of Tregs (−2.030 ± 0.533 for subgroup of SLEDAI ≥ 6, −0.222 ± 0.991 for subgroup of SLEDAI ≥ 5 and −0.056 ± 0.571 for subgroup of SLEDAI ≥ 3, *p* = 0.029).

Lupus nephritis (LN) is the typical major organ manifestation of SLE. In the recruited studies, four studies presented the Tregs data in active LN patients (25, 33, 34, 39) (Table 2). The percentages of Tregs were significantly lower in active LN than those in healthy controls (−2.177 ± 0.972, *p* = 0.025). However, no significant alteration was found when comparing the Treg frequencies of active LN with active SLE patients from the remaining eligible studies (*p* = 0.584).

**Table 2:** Percentages of peripheral Tregs in active LN patients.

**References**	**Treg definition**	**Case**	**Control**	**Tregs in case**	**Tregs in control**
		**(*n*)**	***(n*)**	**(mean ± SD,%)**	**(mean ± SD,%)**
Miyara et al. (25)	CD4+CD25high	23	82	0.533 ± 0.213	1.290 ± 0.380
Tselios et al. (33)	CD4+CD25highFoxP3+	12	20	0.710 ± 0.290	1.490 ± 0.190
Zabinska et al. (34)	CD4+CD25+FoxP3+	40	19	1.073 ± 0.593	3.327 ± 0.519
Mesquita et al. (39)	CD4+CD25+CD127low	17	17	4.548 ± 2.503	3.008 ± 1.511

**Figure 3:**
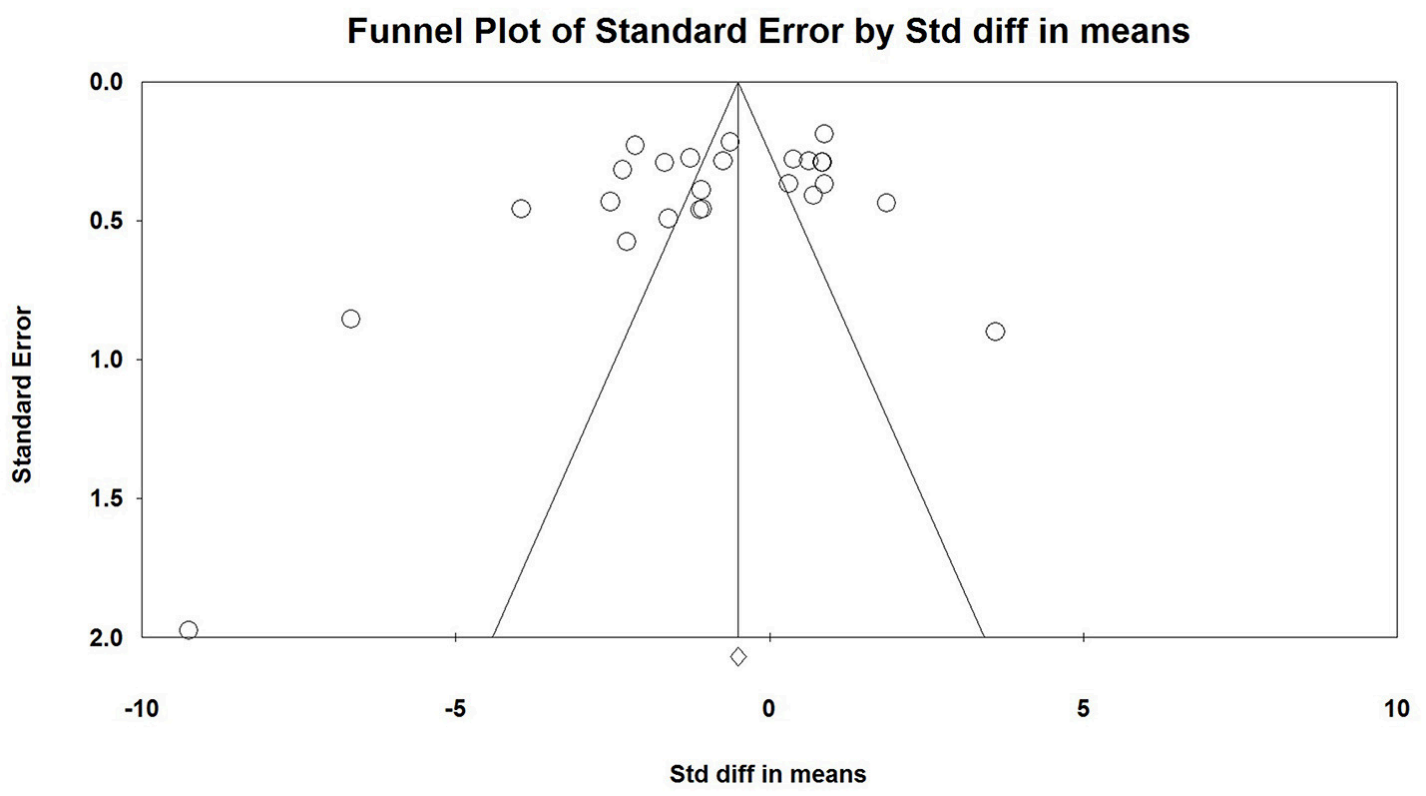
Publication bias analysis using Egger linear regression and Begg rank correlation test.

No publication bias was found by Egger linear regression and Begg rank correlation test (Figure 3).

### Meta-Analysis of Treg Absolute Number Changes in Active SLE

**Figure 4:**
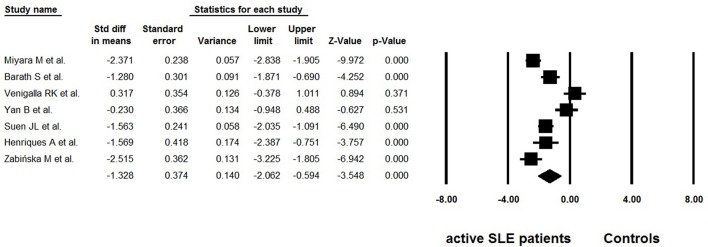
Forest plot of the absolute number changes of Tregs in active SLE patients compared to the controls.

Considering the possible lymphopenia that may occur in SLE patients, and that decreased total number of CD4^+^ T cells may cause calculated “normal” even “increase” in Tregs, some studies simultaneously provided data on the absolute numbers of Tregs. Among the 18 selected studies, 7 reported data on the absolute numbers of Tregs (25, 26, 32, 34–37) (Table 3). Great heterogeneity (*I*^2^ = 90.02) was also found between these studies and the random effects model was used in the meta-analysis. The overall meta-analysis calculated that the absolute numbers of Tregs in active SLE were significantly lower than those in the controls (−1.328 ± 0.374, *p* < 0.001, Figure 4). The potential sources of heterogeneity were determined for subgroup analyses. Subgroup analysis requires that at least two articles be included in every category of the subgroup. As a result, the effects of different gating strategies of Tregs, SLEDAI of enrolled patients, and regions of results were analyzed, but did not identify any significant difference.

**Table 3:** Absolute numbers of peripheral Tregs in active SLE.

**References**	**Treg definition**	**Case**	**Control**	**Tregs in case**	**Tregs in control**
		**(*n*)**	**(*n*)**	**(mean ± SD, cells/mm^**3**^)**	**(mean ± SD, cells/mm^**3**^)**
Miyara et al. (25)	CD4+CD25high	45	82	2.970 ± 2.100	13.510 ± 5.300
Barath et al. (26)	CD4+CD25high	19	41	1.900 ± 1.200	3.900 ± 1.700
Venigalla et al. (36)	CD4+CD25highFoxP3+	14	19	7.967 ± 3.742	6.664 ± 4.359
Yan et al. (37)	CD4+CD25+FoxP3+	15	15	39.810 ± 50.310	48.380 ± 15.920
Suen et al. (32)	CD4+CD25highFoxP3+	58	36	2.330 ± 2.060	5.580 ± 2.110
Henriques et al. (35)	CD25highCD127low/ØFoxP3+	15	15	0.030 ± 0.030	0.070 ± 0.020
Zabinska et al. (34)	CD4+CD25+FoxP3+	40	19	7.487 ± 4.852	21.627 ± 7.007

### Meta-Analysis of Tregs Function in Active SLE

**Figure 5:**
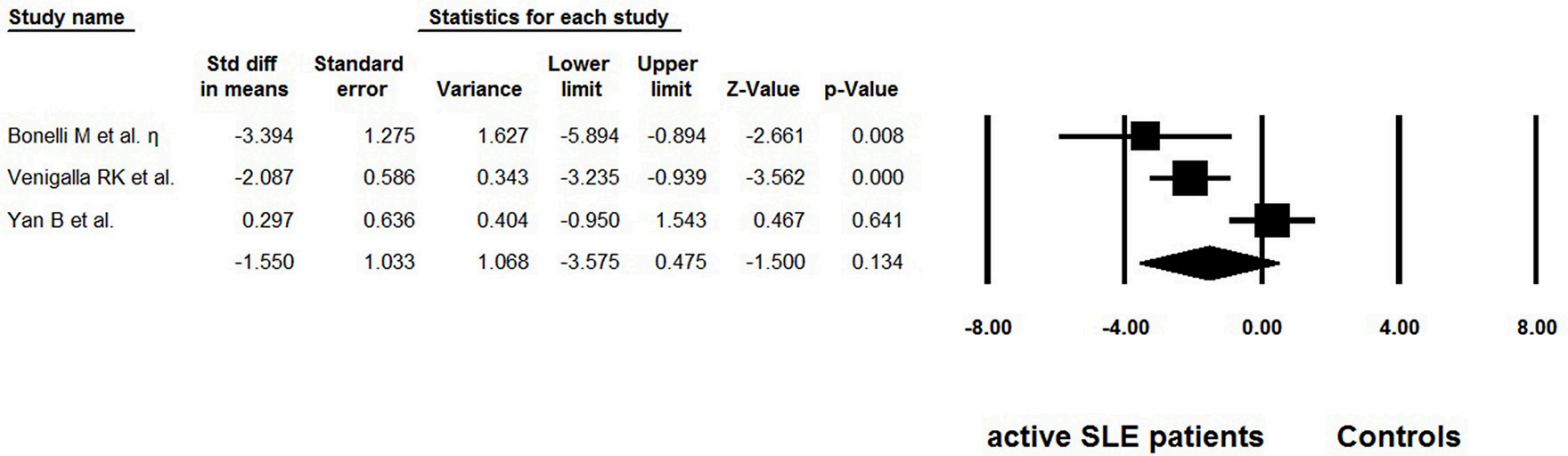
Forest plot of the suppression percentages of Tregs in active SLE patients compared to the controls.

Besides frequency, three studies evaluated the suppressive function of Tregs isolated from active SLE (29, 36, 37) (Table 4). The percentage of suppression was determined as [1– (cpm of coculture/cpm of responder cell alone)] × 100%. We also extracted the data from suppression assays performed independently of the antigen-presenting cells, with suppressor/ responder cell ratio was 1:1. Dysfunctional Tregs were reported in two of the studies (29, 36), whereas suppressive activity of these cells was found to be normal in another study (37). The pooled data did not indicate that function of Tregs was impaired (−1.550 ± 1.033, *p* = 0.475, Figure 5).

**Table 4:** Suppression percentages of Tregs in active SLE.

**References**	**Treg definition**	**Case**	**Control**	**Suppression percentages of Tregs in case**	**Suppression percentages of Tregs in control**
		**(*n*)**	**(*n*)**	**(mean ± SD, %)**	**(mean ± SD, %)**
Venigalla et al. (36)	CD4+CD25highFoxP3+	9	9	53.00 ± 18.00	81.00 ± 6.00
Bonelli et al. (29)	CD4+CD25high	3	3	24.50 ± 21.30	78.00 ± 6.58
Yan et al. (37)	CD4+CD25+FoxP3+	5	5	63.50 ± 17.02	59.42 ± 9.41

### Meta-Analysis of Treg Alterations in Longitudinal Monitoring of Active SLE

We further explored whether Treg percentages would vary within the same individual in relation to different disease status. Longitudinal Treg assessments were performed in two distinct studies (25, 33) (Table 5). The percentage of Tregs was initially measured during disease flare and again following resolution after appropriate treatment. A significant decrease in the percentage of Tregs at remission was found (−2.184 ± 0.499, *p* < 0.001).

**Table 5:** Peripheral Tregs alteration in longitudinal monitoring of active SLE.

**References**	**Treg definition**	**Case**	**Tregs during flare**	**Tregs during remission**
		**(*n*)**	**(mean ± SD, %)**	**(mean ± SD, %)**
Miyara et al. (25)	CD4+CD25high	10	0.39 ± 0.20	1.28 ± 0.39
Tselios et al. (33)	CD4+CD25highFoxP3+	44	0.65 ± 0.27	1.17 ± 0.30

## Discussion

Reduced, unchanged, or even increased frequencies of Tregs have been reported in active SLE patients (25–39, 42–44). Pooled data in the present study showed that both the percentages and absolute numbers of Tregs were significantly lower in patients with active SLE than in the controls (Figures 2, 4). Since the results showed great heterogeneity (*I*^2^> 90), subgroup analysis was subsequently performed to evaluate the possible effect of several factors on the frequencies of Tregs, and the results indicated that different gating strategies of Tregs, diagnostic criteria and different thresholds of SLEDAI chosen for defining active SLE might influence the Treg percentages.

There are several definitions of Tregs with different cell surface markers, and it seems to be a major reason for the discrepancies in the results. Earlier studies relied on CD25 expression for Treg gating. CD4^+^CD25^+^ cells, or CD4^+^CD25^high^ cells were considered to be Tregs. It is now clear that the nuclear transcription factor Foxp3^+^, a critical regulator in the development and function of Tregs, is more specific for gating Tregs, and remains the best protein marker to determine Tregs so far (18). Several lines of evidence have demonstrated that CD127 is an alternative to Foxp3 (19). Co-staining of CD25 and CD127 has been recommended as an efficient way to distinguish Tregs from other T cells and has been widely used to isolate live cells. In the present study, an expected difference was observed between different cell sorting analysis strategies (*p* = 0.022): Tregs gated based only on CD25 staining (CD4^+^CD25^+/high^) (25–29, 31, 42–44) showed lower percentages of Tregs in active SLE than those in controls (−1.672 ± 0.472, *p* < 0.001), whereas normal frequencies were observed when FoxP3^+^ or CD127^low/Ø^ was chosen to define Tregs (−0.269 ± 0.404, *p* = 0.505) (30–39, 42–44). These findings indicated that the Tregs sorting analysis strategy is an important source of heterogeneity and seems to affect the conclusions regarding this T-cell subset.

High-dose glucocorticoid therapy increases the frequency of Tregs in SLE patients (45); in contrast, it has also been reported that Tregs are independent of drug therapy (46). Results of the subgroup analysis on treatment status in the present meta-analysis did not reveal a statistical difference between patients that received drug therapy (25, 26, 29, 30, 32–36, 42–44) and treatment naïve patients (28, 37). Considering that most patients in the current studies received systemic medication, the possible impact of treatment requires to be assessed further.

Our result revealed that the thresholds of SLEDAI chosen to differentiate active SLE from inactive SLE could also result in heterogeneity. The higher threshold of the SLEDAI score, used to define active SLE, tended to achieve a lower percentage of Tregs (−2.030 ± 0.533 for subgroup of SLEDAI ≥ 6, −0.222 ± 0.991 for subgroup of SLEDAI ≥ 5 and −0.056 ± 0.571 for subgroup of SLEDAI ≥ 3, *p* = 0.029). Considering that SLE patients enrolled with a higher threshold of SLEDAI score may have more severe conditions, Treg seem to be negatively correlated with disease activity in SLE.

It should be noted that the diagnostic criteria applied in eligible articles were not consistent. Our subgroup analysis showed that studies that applied the 1997 diagnostic criteria (26–28, 32, 35, 37, 39, 43) calculated lower percentages of Tregs than the studies that applied diagnostic criteria established in 1982 (29, 36, 42, 44). SLE is a heterogeneous disease in which diagnosis is not always easy, and the systems of criteria for diagnosis have undergone changes in recent years. The 1997 ACR classification criteria was an update of the 1982 ACR criteria, and anti-phospholipid antibodies were added to the criteria list. Although these were widely used in clinical practice and clinical research, they have not been validated (47). The impact of different diagnostic criteria on Treg ratios suggest that patients with different diagnostic criteria might have different states of disease. However, the reasons and mechanisms underlying this phenomenon need to be addressed in future.

The percentage of Tregs in total CD4^+^ T cells was the most widely used indicator to evaluate the level of Tregs. Beside percentage, some studies also provided the absolute numbers of Tregs. In a meta-analysis of seven studies (25, 26, 32, 34–37), the overall absolute numbers of Tregs in active SLE were significantly lower than those in the controls (Figure 4), which is consistent with the percentage analysis. However, unlike percentages, the absolute number of Tregs in active SLE does not seem to be affected by gating strategies. In other words, similar decreased trends in the absolute number of Tregs were observed whether Tregs were defined as “CD4^+^CD25^+/high^” or as “Foxp3^+^/CD127^low/Ø^”. Percentages and absolute numbers are both valuable indicators; percentages can indicate whether Tregs are changed with other CD4 cells in a parallel manner, and absolute numbers can indicate the exact number of Tregs. Considering that lymphopenia is frequent in SLE, which may lead to calculated “normal” or even “increased” percentages of Tregs, it is recommended to provide the absolute number as well as percentage data when carrying out related studies in this field.

In addition to frequency and absolute number, functional modifications of Tregs could also lead to breakdown of self-tolerance. In this meta-analysis, the overall suppression percentage of Tregs in active SLE patients showed no abnormality (−1.550 ± 1.033, *p* = 0.475, Figure 5). However, this result should be interpreted with great caution as only three reports were included. Furthermore, this systemic review only included studies related to active SLE, to eliminate the possible heterogeneity resulting from intrinsic differences between active and inactive SLE. Based on this primary objective and eligible criterion, several important studies on this aspect were not included. Some of these studies reported that the Treg function was unimpaired (48, 49), or impaired in some of the SLE patients (50). Most importantly, recent studies showed that IL-21-driven mTOR activation underlies Treg cell dysfunction in SLE (15). Therefore, further research exploring the discrepancy among these important studies is needed.

Meta-analysis of Tregs alteration in longitudinal monitoring of active SLE showed a significant decrease in the percentage of Tregs at remission (−2.184 ± 0.499, *p* < 0.001). This indicates the important role of Tregs in the inflammatory process and implies that the reduced frequency of Tregs may be associated with the development and exacerbation of the disease. However, we cannot draw conclusions regarding their predictive value in assessing disease flare and resolution based on such limited studies, and thus, further well-designed studies are warranted.

Recently, Zhang et al. (51) performed a systemic review on a similar topic, but mainly focused on establishing the alteration of Treg frequency in SLE. However, in our meta-analysis the changes in the frequency and absolute number of Tregs were calculated along with a systemic analysis of the effects of treatment, disease severity, and organ involvement on Tregs. We also evaluated the functional changes of Tregs in active SLE patients. Follow-up studies determining the Treg changes in SLE patients from flare to resolution were also included in our study, to obtain more evidence about the pathogenetic role of Tregs in SLE.

There are some limitations in the present study. Firstly, not all the treatment information is publically available, and we failed to reach the corresponding authors for further information, hindering us from completely investigating the impact of drugs on Treg percentages. Secondly, we must admit that only some of the factors have been found to influence the percentages of Tregs, and the unresolved high heterogeneity requires more studies to be conducted on patients from different backgrounds and disease states to better elucidate the role of Tregs in the disease course.

In summary, our meta-analysis implies that loss of Tregs may play a role in SLE pathogenesis. The differences among studies including gating strategies for Tregs, diagnostic criteria for SLE, and thresholds of SLEDAI chosen to differentiate active and inactive SLE, seem to be the major reasons for discrepancies in published results on active SLE. Our study represents an additional piece to help solve the puzzle of contradictory results on Tregs in SLE and shed new light on the therapeutic potential of Tregs in this field.

## Author Contributions

GW: study design. WL and CD: data collection. WL, CD, and HY: statistical analysis. WL: paper writing. GW: paper revision. All authors approved the submitted version of the manuscript.

### Conflict of Interest Statement

The authors declare that the research was conducted in the absence of any commercial or financial relationships that could be construed as a potential conflict of interest.
